# Social Determinants of Protein-Energy Malnutrition: Need to Attack the Causes of the Causes

**DOI:** 10.3329/jhpn.v28i3.5562

**Published:** 2010-06

**Authors:** Gagan Gurung

**Affiliations:** Save the Children, Nepal Family Health Programme, Kathmandu, Nepal

Sir,

At the current mortality levels, 1 in every 21 Nepalese children dies before reaching the age of one year, while 1 in every 16 children does not survive to the fifth birthday (1). Nepal has achieved a spectacular success in reducing child mortality in recent decades; however, much must be done to achieve the Millennium Development Goal (MDG). Among different factors, the nutritional status of children plays a key role in determining mortality and morbidity of children.

Malnutrition is still a serious threat to child development and survival in Nepal. Protein-energy malnutrition (PEM) and micronutrient deficiency (iodine, iron, and vitamin A deficiency) are the most common forms. Various programmes, such as control of iodine-deficiency disorders and control of vitamin A deficiency, have been undertaken to tackle the problem arisen from deficiency of micronutrients (2). Some programmes, such as the National Vitamin A Programme, have claimed to be successful public-health interventions in Nepal. Similarly, other micronutrient initiatives by different non-governmental organizations are underway. However, the Government of Nepal does not have any strong and effective programmes to tackle the pervasive problem of macronutrients, i.e. PEM.

Before 1980, most attention in developing countries was focused on PEM but the attention of the nutrition community and resources of donors were slowly attracted by the glamour of micronutrients, a largely technical, and often top-down, solution (close to a ‘quick fix’ magic bullet) (3).

The Nepal Demographic and Health Survey (NDHS) 2006 showed that 49% of children aged less than five years were affected by stunting, 39% were underweight, and 13% were wasted (1). To make the matter worse, the situation of PEM is not evenly distributed across the country. Rural area, far and mid-western region, and poor and uneducated population are badly affected by PEM. When compared with the NDHS 2001, some improvements in the nutritional status of children has been observed in the last five years. The percentage of children stunted fell by 14 percentage points from 57% in 2001 to 49% in 2006. Underweight has declined only slightly (from 43% to 39%), and wasting has risen from 11% in 2001 to 13% in 2006. One of the indicators of MDG in Nepal is to reduce the prevalence of underweight children aged less than five years from 57% in 1990 by half to 29% in 2015 (4). However, the above results indicate that, achieving the MDG of a 50% reduction in the prevalence of underweight children aged less than five years by 2015 continues to be a challenge.

The current strategies of the Government of Nepal to control PEM include awareness-raising and nutrition education to the community, growth monitoring, behavioural change and communication, and nutritional rehabilitation. In other words, the response of the Government to tackle the problem is confined to a traditional approach of preventive and promotive care. The mere screening of growth of the children, educational action to change behaviours, and the suggestion of offering nutritious food to poor mothers/caretakers do not solve the problem of PEM. Food and nutrition education, in the presence of widespread food shortages, ends up in teaching people to eat what they cannot afford or do not have and, thus, has only limited potential. It reflects an attitude, such as ‘keep them poor, but teach them’ (5). The crux of the problem is poverty, food insecurity, skewed land distribution, etc. These are the social determinants of PEM. A mere behavioural and medical approach would only deal with the proximal cause but not with the distal and the causes of the causes of PEM. PEM is not a simple problem with a simple solution. It results from the complex interplay of social and biomedical factors. Hence, PEM is a social disease (6). A holistic and multi-pronged approach is needed with a strong socioeconomic policy reform by the Government. The policy of the Government should focus on addressing the underlying causes of malnutrition, with particular attention to the inequities in income distribution, the need for greater agricultural productivity, necessary improvement in the purchasing power of families, and on food conservation, processing, marketing and pricing mechanisms.

## References

[1] Nepal (2007). Ministry of Health and Population. Nepal demographic and health survey 2006.

[2] Nepal (2007). Ministry of Health and Population. Department of Health Services. Annual report, 2006/2007.

[3] Schuftan C, Ramalingaswami V, Levinson FJ (1998). Micronutrient deficiencies and protein-energy malnutrition. Lancet.

[4] Nepal (2005). National Planning Commission. Nepal Millennium Development Goal: progress report 2005.

[5] Schuftan C The political economy of ill health and malnutrition.

[6] Park K (2005). Park's Textbook of preventive and socialmedicine, 18th ed..

